# Capsulolabral Reconstruction During the Open Latarjet Procedure

**DOI:** 10.1016/j.eats.2021.07.017

**Published:** 2021-10-06

**Authors:** Marko Nabergoj, Matthias Zumstein, Patrick J. Denard, Philippe Collin, Sean Wei Loong Ho, Sidi Wang, Alexandre Lädermann

**Affiliations:** a Valdoltra Orthopaedic Hospital, Ankaran, Slovenia; b Faculty of Medicine, University of Ljubljana, Ljubljana, Slovenia; c Shoulder, Elbow and Orthopaedic Sports Medicine, Orthopädie Sonnenhof, Bern, Switzerland; d Department of Orthopaedic & Rehabilitation, Oregon Health & Science University, Portland, Oregon, U.S.A.; e Centre Hospitalier Privé Saint-Grégoire (Vivalto Santé), Saint-Grégoire, France; f Department of Orthopaedic Surgery, Tan Tock Seng Hospital, Singapore; g Research Department, La Tour Hospital, 1217 Meyrin, Switzerland; h Faculty of Medicine, University of Geneva; i Division of Orthopaedics and Trauma Surgery, Department of Surgery, Geneva University Hospitals, Geneva, Switzerland

## Abstract

Surgical treatment of anterior glenohumeral joint instability can be challenging and carries the inherent risk of recurrent instability, dislocation arthropathy, and postoperative loss of external rotation. In the current manuscript, a technique for combined reconstruction of anterior labrum and capsule, with concomitant reduction of the humeral head during anterior capsule reconstruction in open Latarjet procedure, is presented. Analogous to other techniques, the coracoid graft is fixed on the anteroinferior part of the glenoid between 3 and 5 o'clock. However, for this technique, reattachment of the labrum is performed between the native glenoid and the bone graft. Additionally, during the reconstruction of the anterior capsule on the coracoacromial ligament, while the operated arm is held in external rotation to avoid the postoperative rotational deficit, the humeral head is reduced posteriorly in the center of the glenoid during adduction, slight anterior forward flexion, and a posterior lever push. By doing so, the inherent theoretical risks of persistent instability and dislocation arthropathy are believed to be decreased. Further studies are needed to clarify the long-term consequences of this surgical technique in the clinical setting.

The Latarjet procedure^1^ has been shown to be safe and reliable in managing cases of anteroinferior glenohumeral instability with or without significant glenoid bone loss.^2^^,^^3^ This technique is very efficient, as long-term follow-up studies of more than 10 years reported a redislocation rate of 3.2% and revision rate of 3.7%, whereas 1.6% of patients underwent revision because of recurrence.^4^ However, persistent apprehension of patients has been reported in up to 51% of the cases despite a clinically stable joint.^5^ Shoulder dislocation causes damage to the capsuloligamentous complex in 52% of cases^6^ and the glenoid labrum in 73% of cases.^7^ The plastic deformation of these structures becomes progressively worse with subsequent episodes.^8^^,^^9^ Capsular redundancy also has been recognized as a risk factor for ongoing apprehension after surgical stabilization.^10^ Ropars et al.^11^ found a significantly decreased apprehension in patients with associated capsulorrhaphy compared with patients with Latarjet and no capsular reconstruction.

Numerous different techniques of capsulolabral management have been described, from resection of the labrum and capsule,^12^ 2-flap elevation, T- or L-shaped incision into the capsule, and repair of the anterior capsule to the glenoid rim (extra-articular bone graft) and the grafted bone (intra-articular bone graft).^13^ However, no article has described the reduction of the humeral head while tightening the knots during the capsule reconstruction.^11^^,^^13^, ^14^, ^15^, ^16^, ^17^, ^18^, ^19^ The reconstruction of both labral and anterior capsular structures has been described with the arthroscopic Latarjet technique^20^^,^^21^ but rarely in the open procedure.^22^ This Technical Note aims to describe our preferred surgical technique of capsulolabral management during open Latarjet procedure.

## Surgical Technique (With Video Illustration)

### Preoperative Patient Positioning

The open procedure is performed with the patient under an interscalene block and general anesthesia in a semi–beach-chair position. Before prepping and draping of the arm begins, the shoulder is evaluated for instability.

### Approach

**Fig 1:**
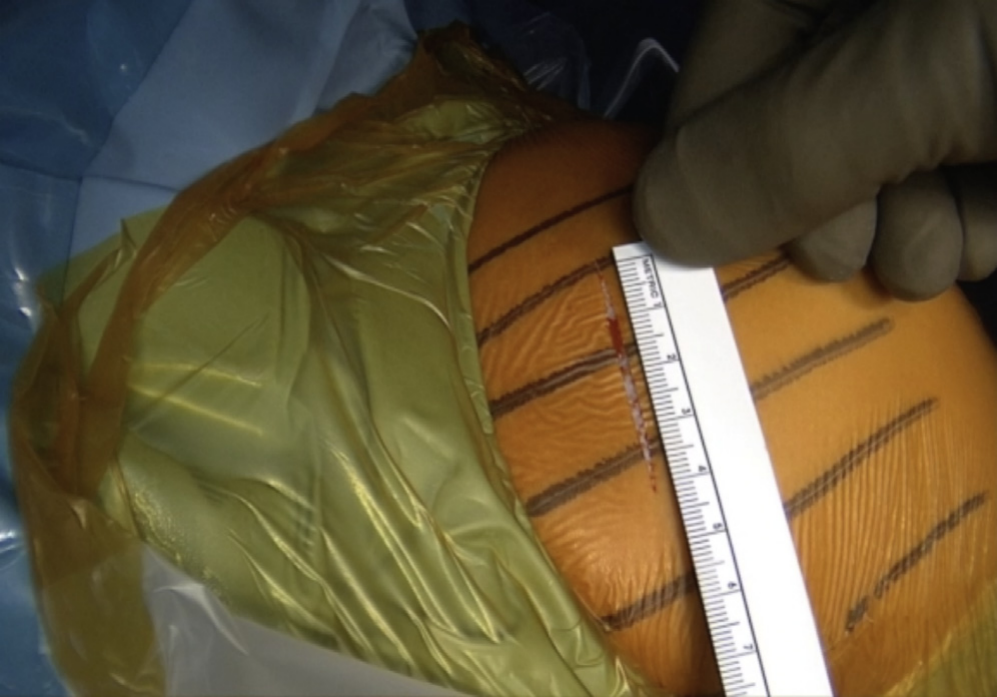
Left shoulder of a patient placed in a semi–beach-chair position. The tip of the coracoid process is palpated, and the incision is performed under the tip of the coracoid process extending 4 to 5 cm distally.

**Fig 2:**
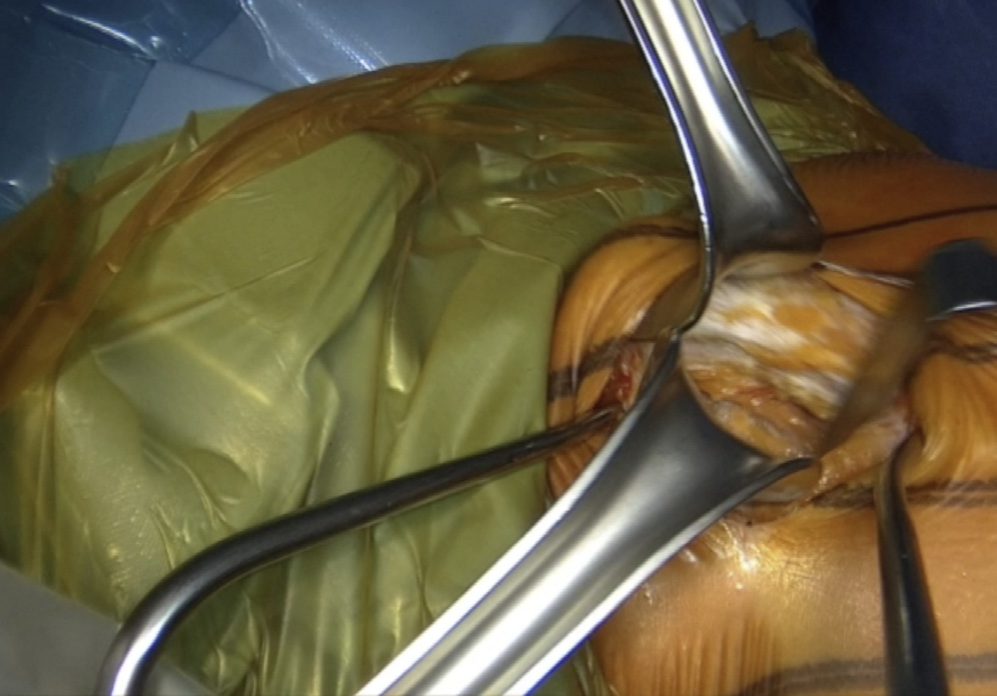
Left shoulder of a patient placed in a semi–beach-chair position. The dissection begins at the level of the Mohrenheim fossa, a triangular region just inferior to the clavicle, between the deltoid and pectoralis major muscles, which do not contain neurovascular structures. The deltopectoral interval is then opened bluntly with 2 Richardson retractors.

**Fig 3:**
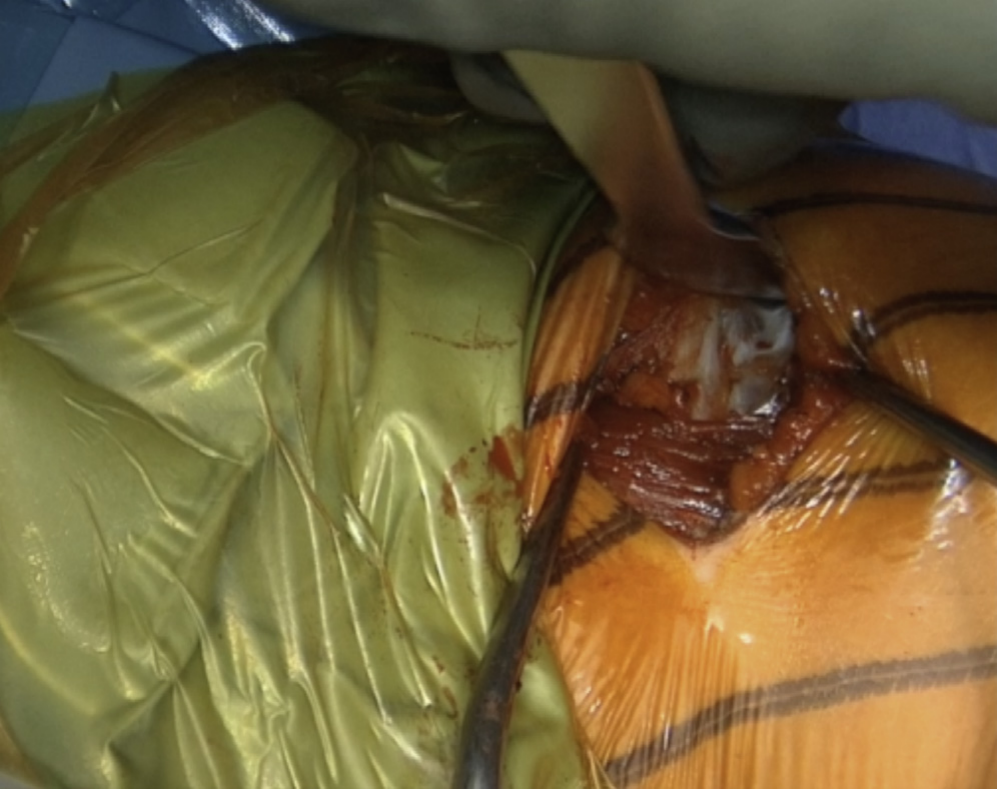
Left shoulder of a patient placed in a semi–beach-chair position. The whole coracoid process with the insertion of pectoralis minor, coracoacromial, and the conjoined tendon is exposed by placing the Hohmann retractor on its tip.

**Fig 4:**
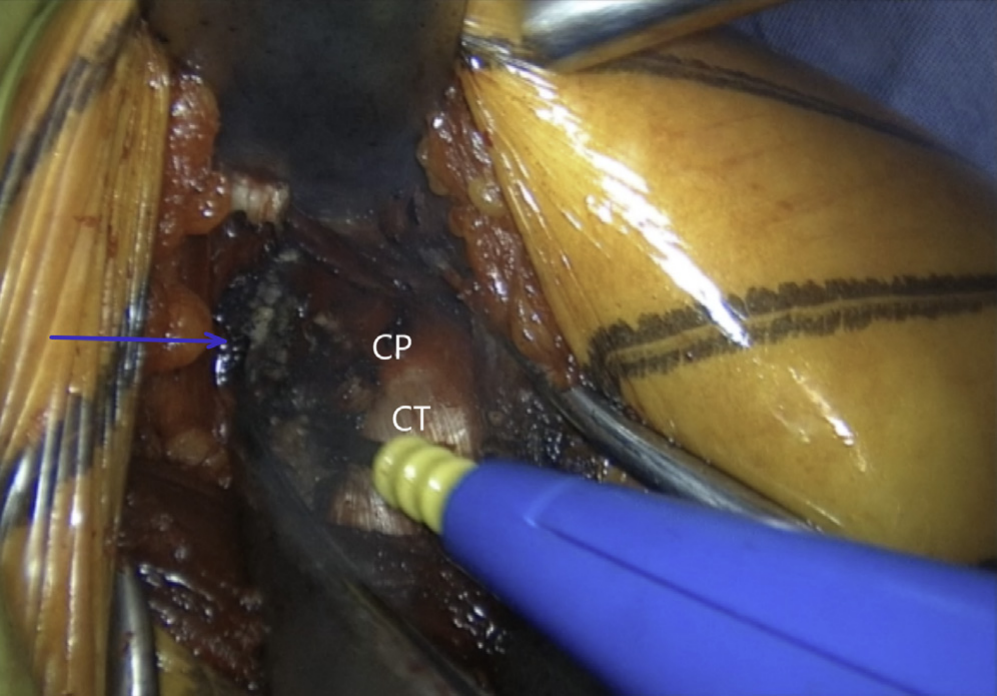
Left shoulder of a patient placed in a semi–beach-chair position. Pectoralis minor (blue arrow) is released from the coracoid process (CP) while the arm is in adduction and internal rotation. (CT, conjoined tendon.)

**Fig 5:**
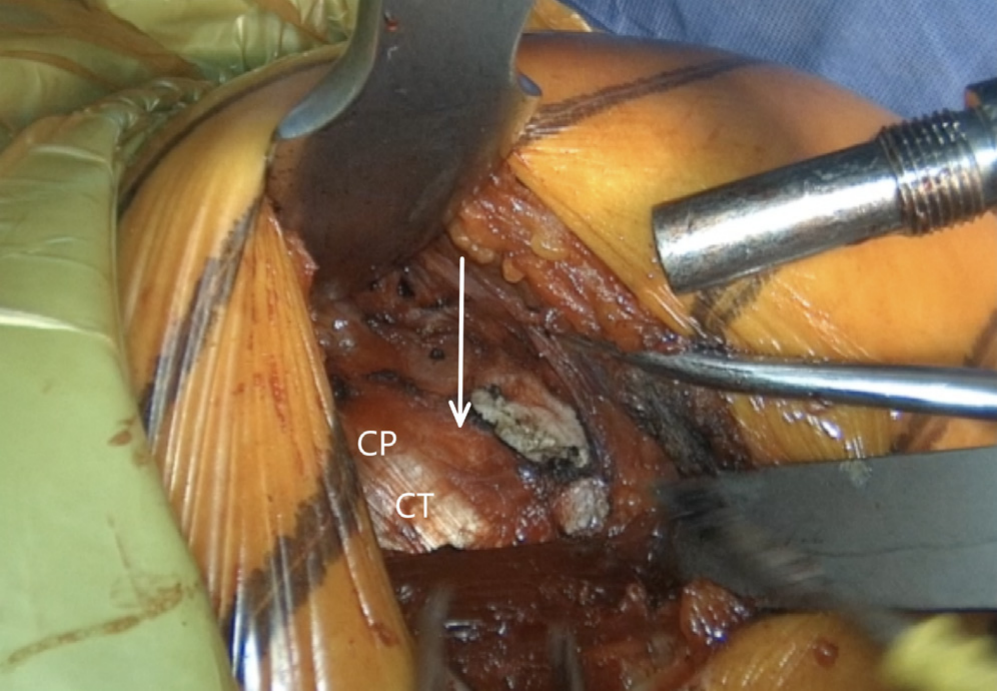
Left shoulder of a patient placed in a semi–beach-chair position. The coracoacromial ligament (white arrow) is released 1.5 cm laterally from its attachment on the coracoid process (CP). The arm is abducted and externally rotated for 90° to improve its visualization. (CT, conjoined tendon.)

The incision is performed under the tip of the coracoid process extending 4 to 5 cm distally (Fig 1 and Video 1). The dissection begins at the level of the Mohrenheim fossa, a triangular region just inferior to the clavicle, between the deltoid and pectoralis major muscles which do not contain neurovascular structures. The deltopectoral interval is then opened bluntly with 2 Richardson retractors, letting the cephalic vein medially (Fig 2 and Video 1). The Gelpi retractor is placed deep in the approach, whereas the cephalic vein is retracted laterally. The whole coracoid process with the insertion of pectoralis minor, coracoacromial ligament, and the conjoined tendon is exposed by placing the Hohmann retractor on its tip (Fig 3 and Video 1). The pectoralis minor is released from the coracoid process with electrocautery while the arm is internally rotated and adducted (Fig 4 and Video 1). The upper limb is abducted and fully externally rotated to improve the coracoacromial ligament visualization, which is then released approximately 1.5 cm laterally from its attachment (Fig 5 and Video 1).

### Coracoid Graft Harvest and Preparation

**Fig 6:**
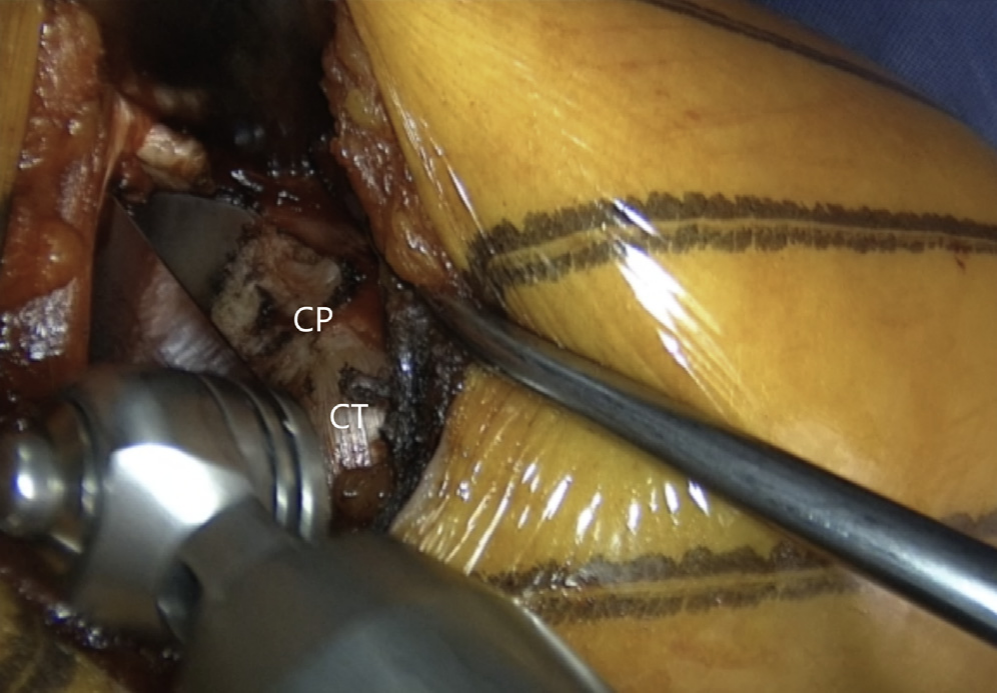
Left shoulder of a patient placed in a semi–beach-chair position. A 90° angled saw blade is used for coracoid osteotomy, which is performed at the base of the coracoid process (CP) as far back as possible, however, still in front of the coracoclavicular ligaments. (CT, conjoined tendon.)

**Fig 7:**
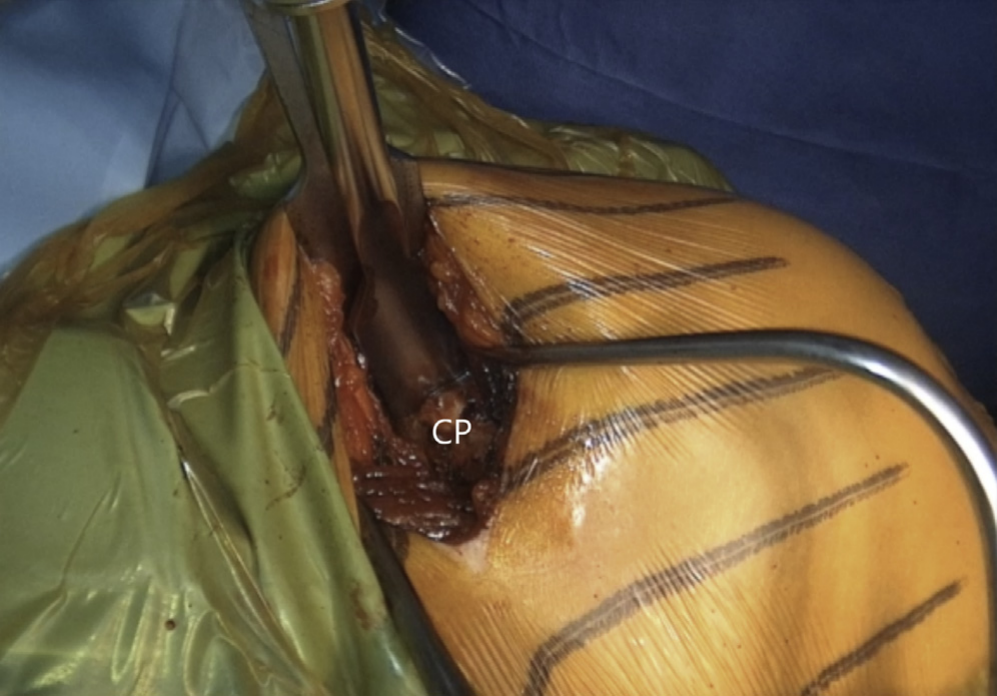
Left shoulder of a patient placed in a semi–beach-chair position. The coracoid osteotomy is meticulously finished with a chisel. (CP, coracoid process.)

**Fig 8:**
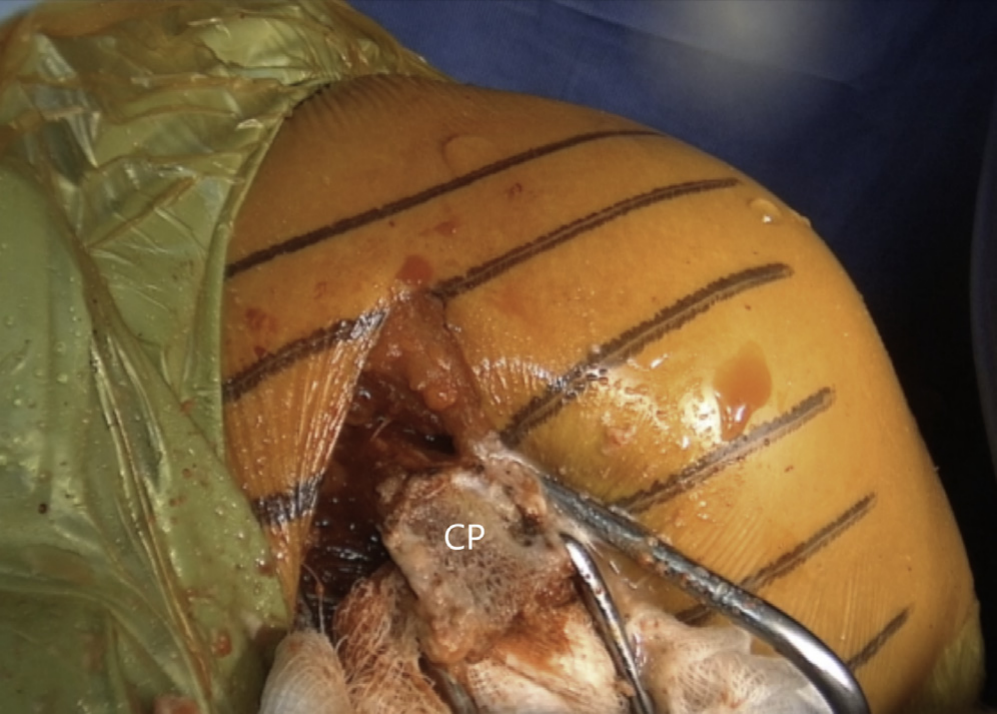
Left shoulder of a patient placed in a semi–beach-chair position. The undersurface of the coracoid process (CP) is flattened and slightly decorticated with a saw blade to create a healthy bleeding surface that will precisely conform to the later prepared anterior glenoid.

**Fig 9:**
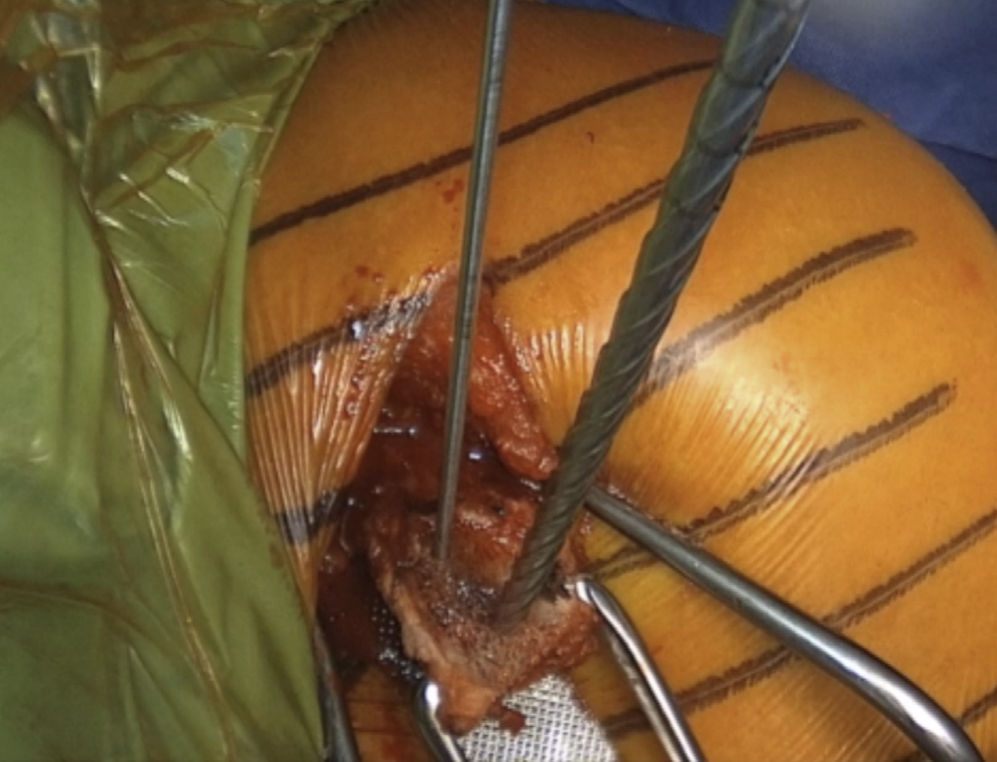
Left shoulder of a patient placed in a semi–beach-chair position. Two 4-mm holes for screw fixation are predrilled perpendicularly and centrally in the coracoid graft, 1 cm apart, and 8 to 9 mm laterally from the insertion of the coracoacromial.

A 90° angled saw blade is used to perform a coracoid process osteotomy at its base as far back as possible but still just anterior to the coracoclavicular ligament, starting superomedialy and proceeding inferolateraly (Fig 6 and Video 1). When the coracoid process gets loose, a chisel is meticulously used to complete the osteotomy (Fig 7 and Video 1). The coracoid process is rotated for 180° while being held with a grasper. It is attentively released until the muscle belly is uncovered to be easily and safely manipulated. The coracoid process should not be placed outside the surgical field to avoid tension in musculocutaneous nerve neuropraxia. Its undersurface is flattened and slightly decorticated with a saw blade to create a healthy bleeding surface that will precisely conform to the later prepared anterior glenoid (Fig 8 and Video 1). The two 4-mm holes for screw fixation are drilled equally distant from the base and the tip, 1 cm apart and 8 to 9 mm laterally from the insertion of the coracoacromial (Fig 9 and Video 1). It is essential that the holes are drilled perpendicularly to the surface and centrally to the graft. There are 2 options for labral fixation, either by transosseous coracoid fixation or by fixation with anchors at the later medial coracoid–glenoid edge. If the surgeon chooses the transosseous coracoid fixation, 2 holes for later labral fixation are predrilled with a K-wire on the lateral coracoid process bony rim where the coracoacromial inserts, but so that they are placed bellow it and do not pass it. A nonresorbable suture is shuttled through each of them. The coracoid process is retracted medially with the pectoralis major muscle.

### Glenoid Exposure and Preparation

**Fig 10:**
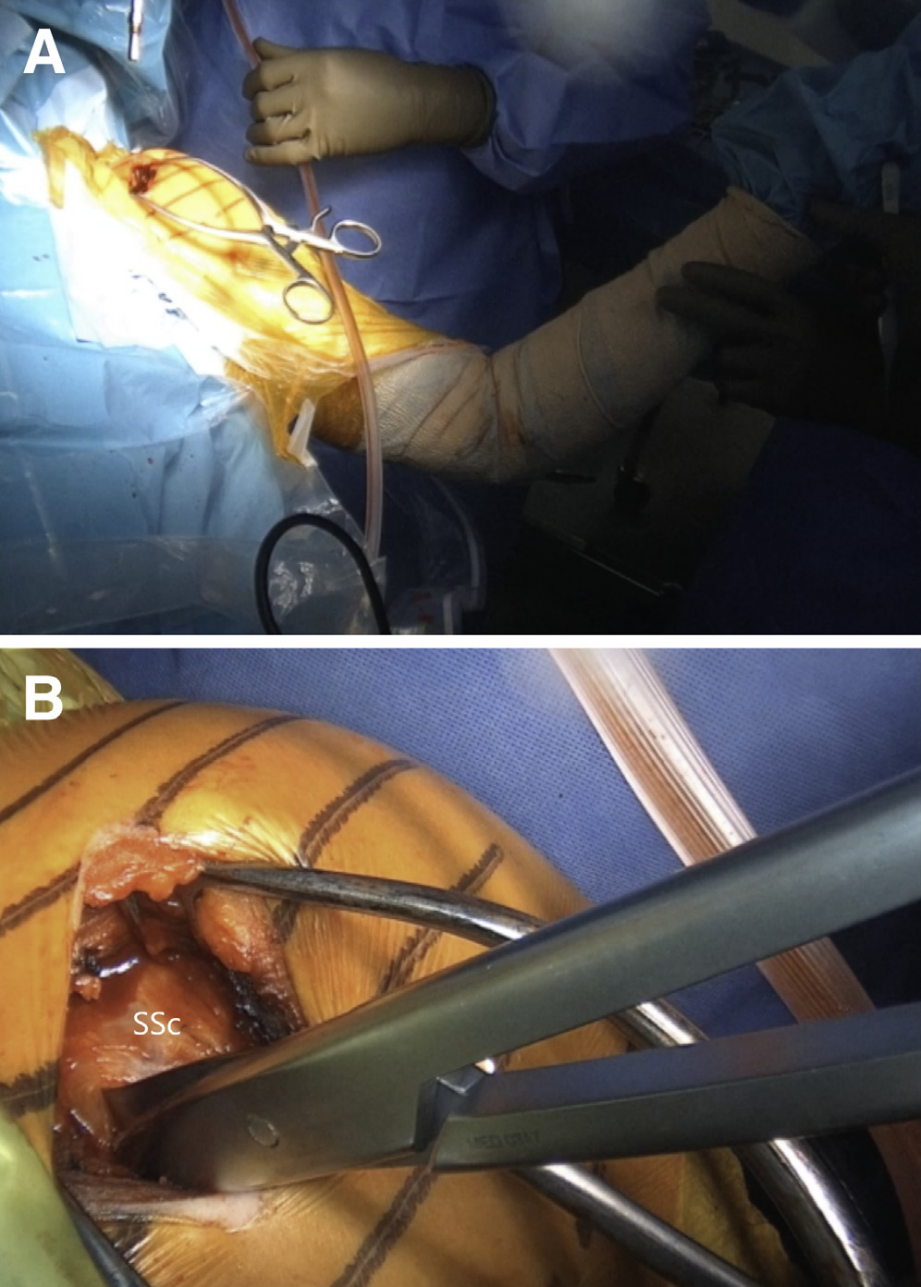
(A) Left upper extremity of a patient placed in a semi–beach-chair position. The arm is placed in abduction and external rotation. (B) Left shoulder of a patient placed in a semi beach-chair position. The subscapularis split between the upper two thirds and lower one third of the subscapularis (SSc) is performed by sharply introducing horizontally placed scissors through the subscapularis muscle toward the anterior glenoid neck. Then they are rotated for 90°.

**Fig 11:**
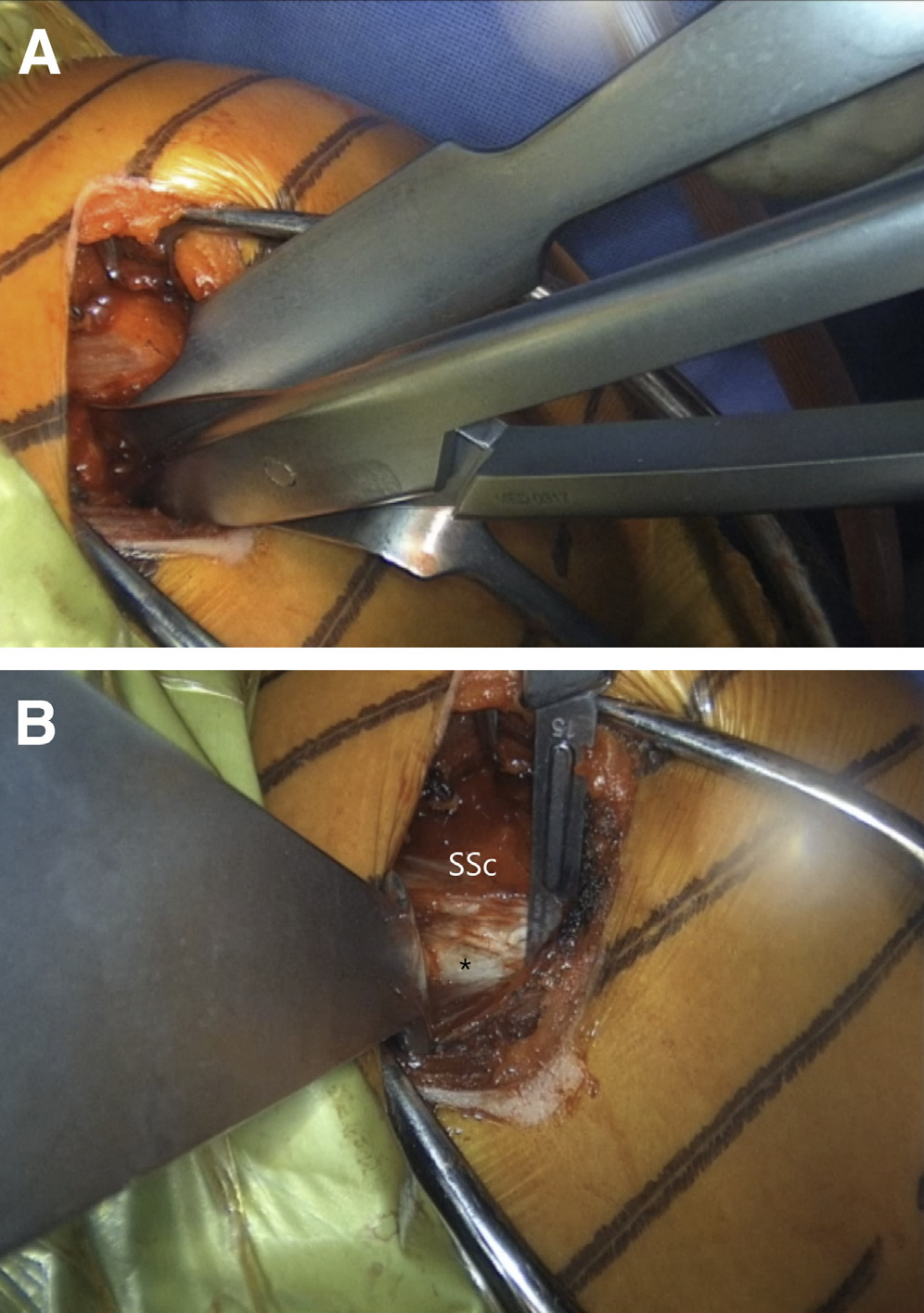
Left shoulder of a patient placed in a semi–beach-chair position. (A) The blades of the scissors are extended to widen the split, while a Hohmann retractor is placed between the blades on the medial side of the AGN. (B) The split is additionally increased with a No. 15 blade. ∗ indicates the anterior capsule. (AGN, anterior glenoid neck; SSc, subscapularis.)

**Fig 12:**
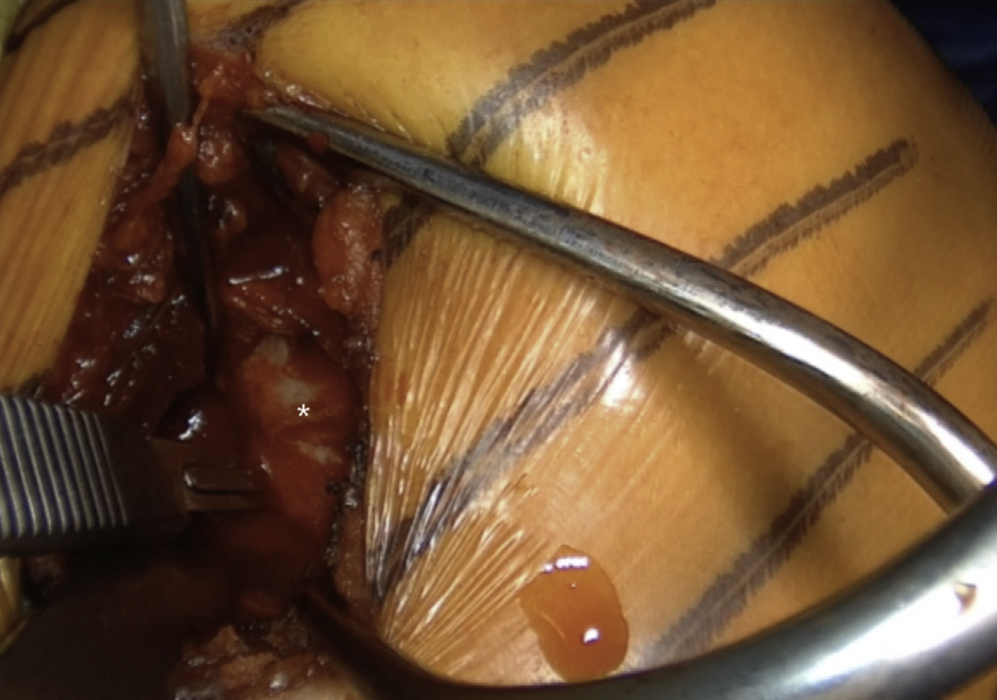
Left shoulder of a patient placed in a semi–beach-chair position. The exact location of the glenohumeral joint is exposed by reducing the anteriorly dislocated humeral head, and a vertical incision is performed in an inferior to a superior direction to protect the axillary nerve. ∗ indicates the anterior capsule.

**Fig 13:**
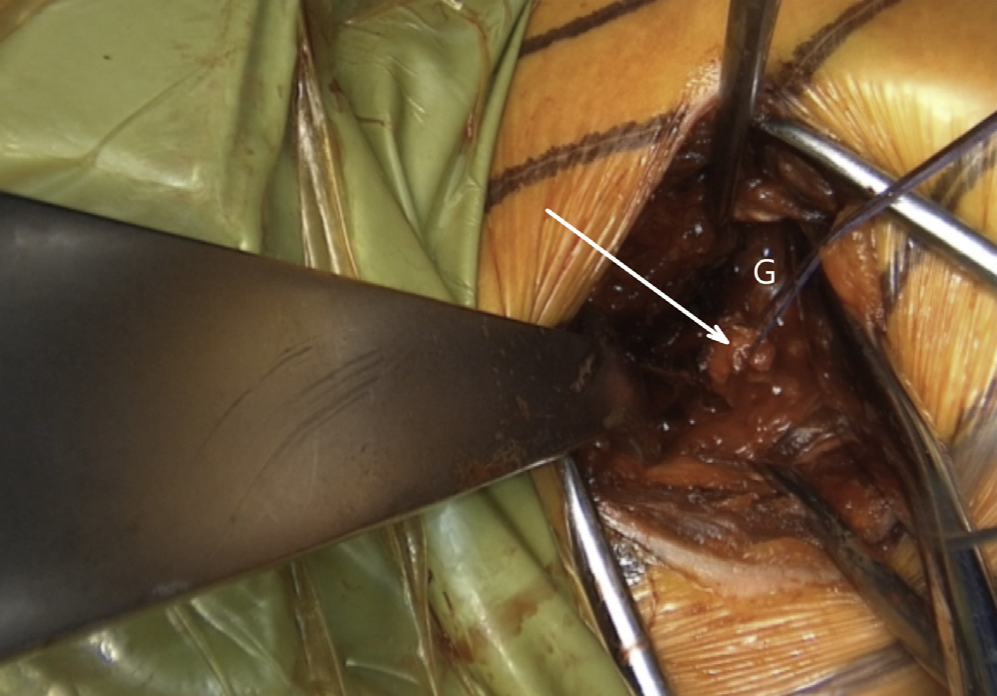
Left shoulder of a patient placed in a semi–beach-chair position. The labrum is horizontally released at the level of 3-o’clock position, and the release is extended inferiorly until the 5-o’clock position. A resorbable suture is passed through the superior half of the released labrum (white arrow). Afterward, another suture is passed through the inferior half of the released labrum for later labral reconstruction. (G, glenoid.)

**Fig 14:**
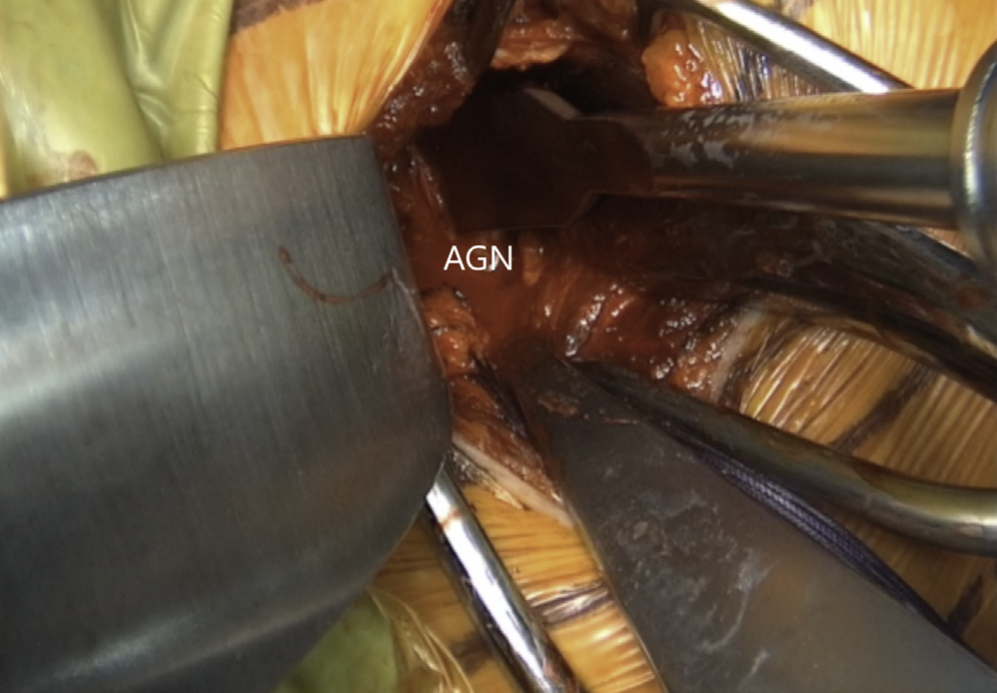
Left shoulder of a patient placed in a semi–beach-chair position. A curved osteotome is used to lightly decorticate the AGN from 3- to 5-o’clock position to a healthy and bleeding flat bone bed. (AGN, anterior glenoid neck.)

**Fig 15:**
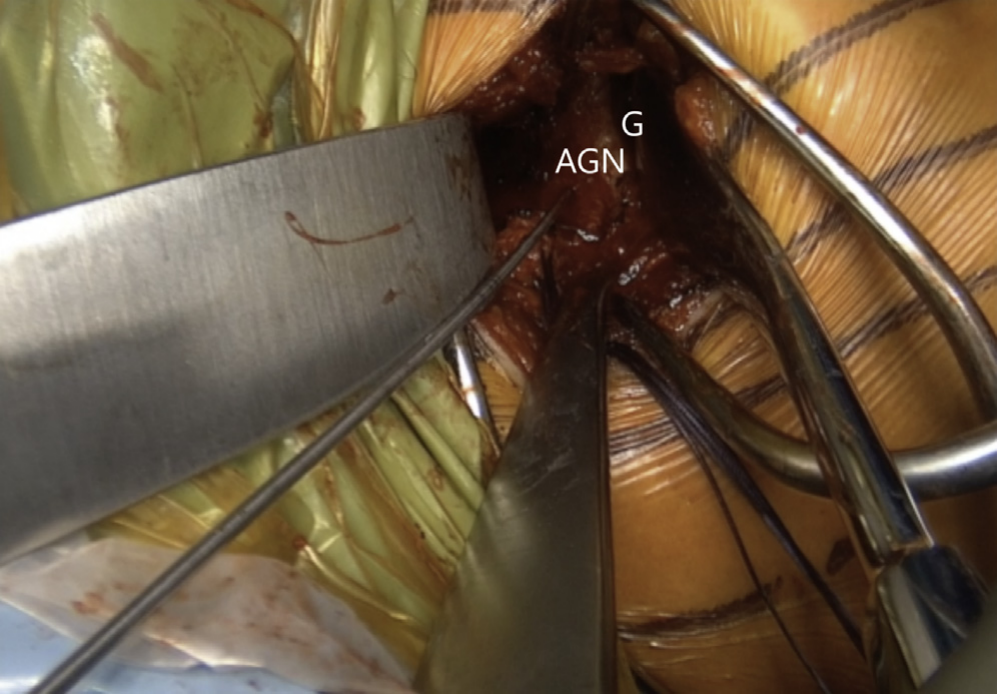
Left shoulder of a patient placed in a semi–beach-chair position. The inferior pilot hole aimed less than 10° away from the glenoid articular surface is drilled first with a K-wire and then with a 2.75-mm cannulated drill in the AGN, located 8 mm from the anterior glenoid. (AGN, anterior glenoid neck; G, glenoid.)

The arm is placed in abduction and external rotation and the subscapularis split between the upper two thirds and lower one third of the subscapularis is performed by sharply introducing horizontally placed scissors towards the anterior glenoid neck. Then, they are rotated for 90° (Fig 10 and Video 1). Their blades are extended to widen the split, while a Hohmann retractor is placed between the blades on the medial side of anterior glenoid neck. The division is additionally increased with a No. 15 blade (Fig 11 and Video 1). The superior and inferior parts of the subscapularis are held apart by 2 Gelpi retractors, one placed superficially and one deeper, while a Hohmann retractor is placed on the inferior aspect of the glenoid neck. The glenohumeral joint's exact location is exposed by reducing the anteriorly dislocated humeral head, and a vertical incision is performed (Fig 12 and Video 1). A Trillat instrument is introduced in the joint to slightly posteriorly subluxate the humeral head to get a better view of the anterior labrum, and a wide glenoid retractor is exchanged with the Hohmann retractor on the medial side of the anterior glenoid to improve the visualization of the anterior glenoid. The labrum is horizontally released at the level of 3-o’clock position, and the release is extended inferiorly until the 5 o'clock position. Two nonresorbable sutures are passed through the superior and inferior half of the released labrum for later labral reconstruction (Fig 13 and Video 1). A curved osteotome is used to slightly decorticate the anterior glenoid neck from the 3-o’clock to 5-o’clock position to a healthy and bleeding flat bone bed (Fig 14 and Video 1). The inferior hole aimed less than 10° away from the glenoid articular surface is predrilled with a 2.75-mm cannulated drill in the anterior glenoid neck, located 8 to 9 mm from the anterior glenoid (Fig 15 and Video 1).

### Coracoid Process Transfer

**Fig 16:**
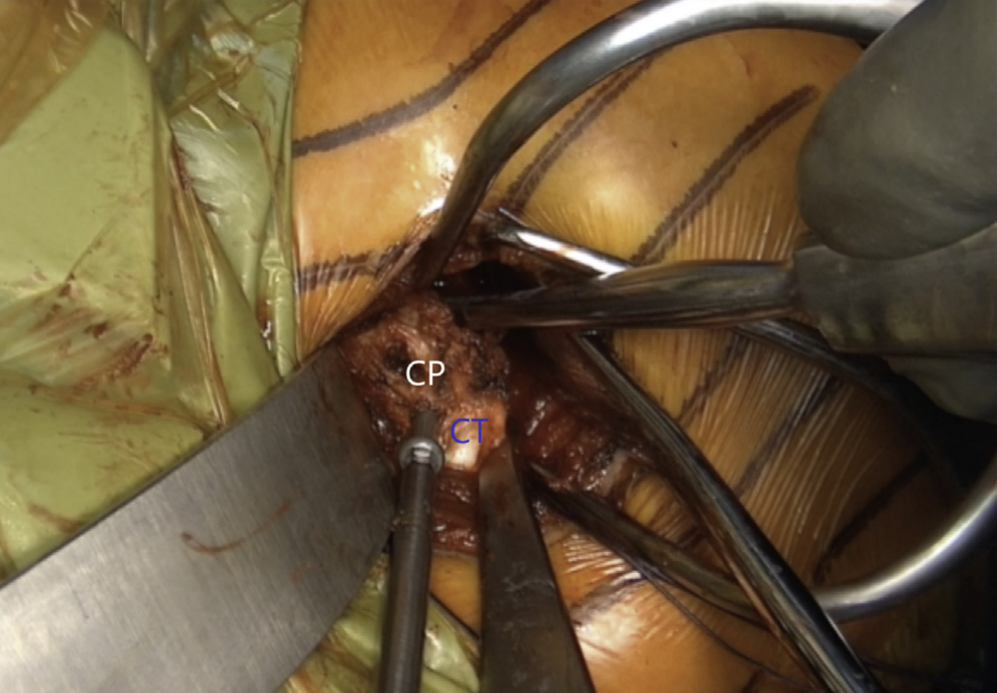
Left shoulder of a patient placed in a semi beach-chair position. The coracoid process (CP) is placed at the prepared AGN surface. First, the inferior screw is introduced for preliminary fixation of the coracoid process so that the graft can still slightly rotate around the screw. (CT, conjoined tendon.)

**Fig 17:**
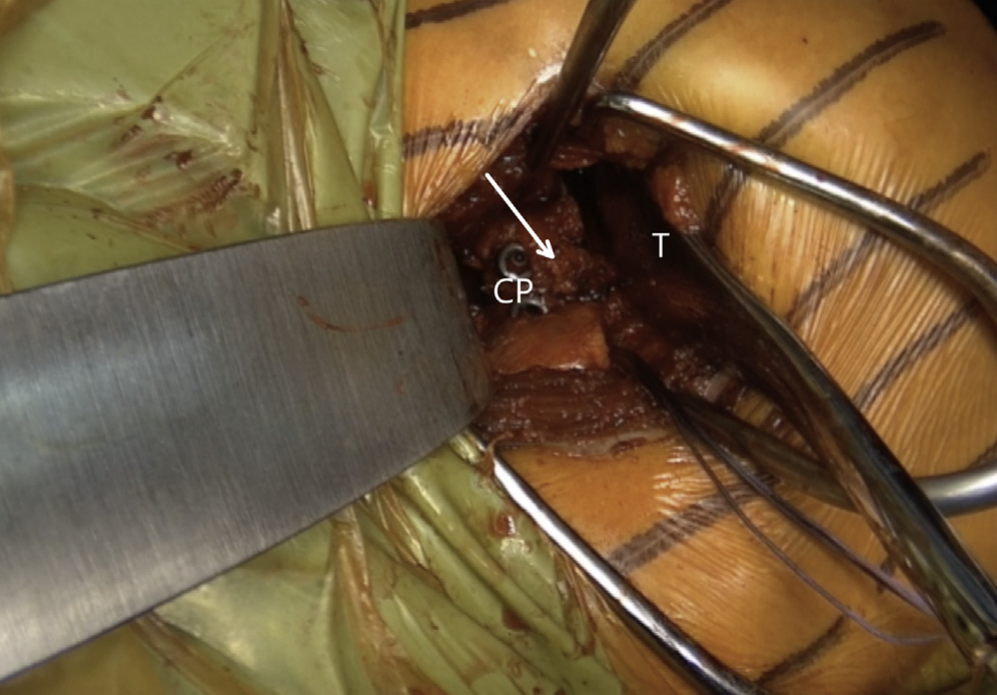
Left shoulder of a patient placed in a semi beach-chair position. A Trillat retractor (T) is used to place the superior part of the coracoid process flush with the glenoid face. The superior hole is drilled with a 2.75-mm cannulated drill in the AGN, the length is measured, and the screw is introduced but not fully tightened. White arrow indicates the coracoacromial ligament. (CP, coracoid process.)

**Fig 18:**
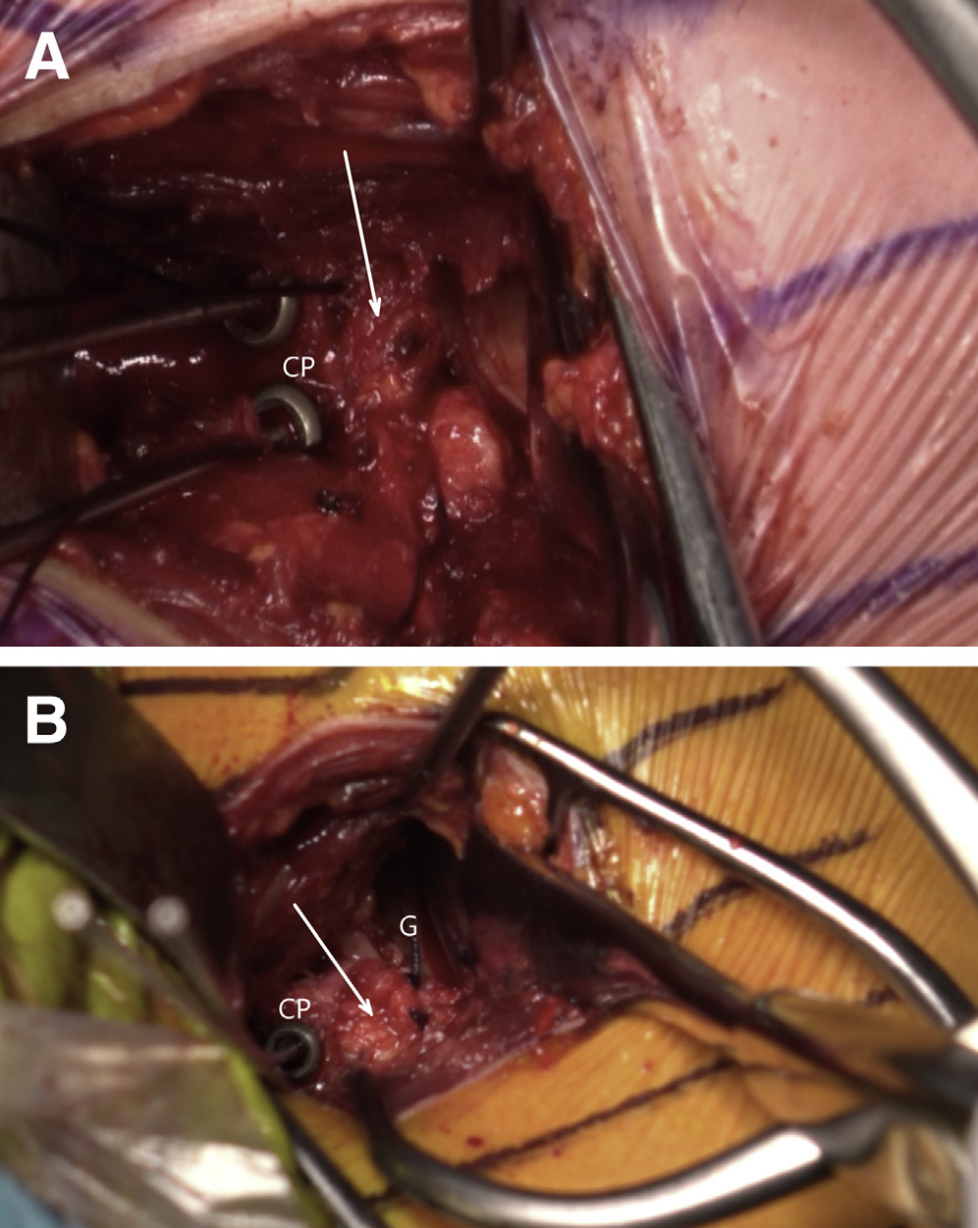
Left shoulder of a patient placed in a semi beach-chair position. Reattachment of the labrum (white arrow) on the coracoid process (CP) remains to be done (A). The labral reattachment (white arrow) has been performed (B). (G, glenoid.)

The coracoid process is retrieved and the 2 sutures that were previously placed through the labrum are inserted through the 2 predrilled graft holes if the transosseous labral fixation is underway. The coracoid process is placed at the prepared anterior glenoid neck surface. A K-wire is passed through the lower predrilled coracoid and glenoid hole to position the coracoid process on the anterior glenoid neck. The screw length is measured, and the screw is introduced for preliminary fixation (Fig 16 and Video 1). A thin Darrach retractor is used to place the superior part of the coracoid process flush with the glenoid face. Afterward, the superior hole is drilled with a 2.75-mm cannulated drill in the anterior glenoid neck, the length is measured, and the screw is introduced but not fully tightened (Fig 17 and Video 1). The anterior labrum is fixed on the coracoid process by tightening the knots of the sutures passing through the labrum (Fig 18 and Video 1). Then, the coracoid is fully fixed by completely tightening the 2 partially threaded 4.0-mm cancellous screws. This accomplishes an excellent compression between the coracoid process and the anterior glenoid neck due to the lag-by-design technique. If, however, the surgeon chooses an anchor technique, approximately 2 anchors are placed at the medial coracoid-glenoid edge, and fixation of the labrum is performed.

### Capsule and Subscapularis Repair

**Fig 19:**
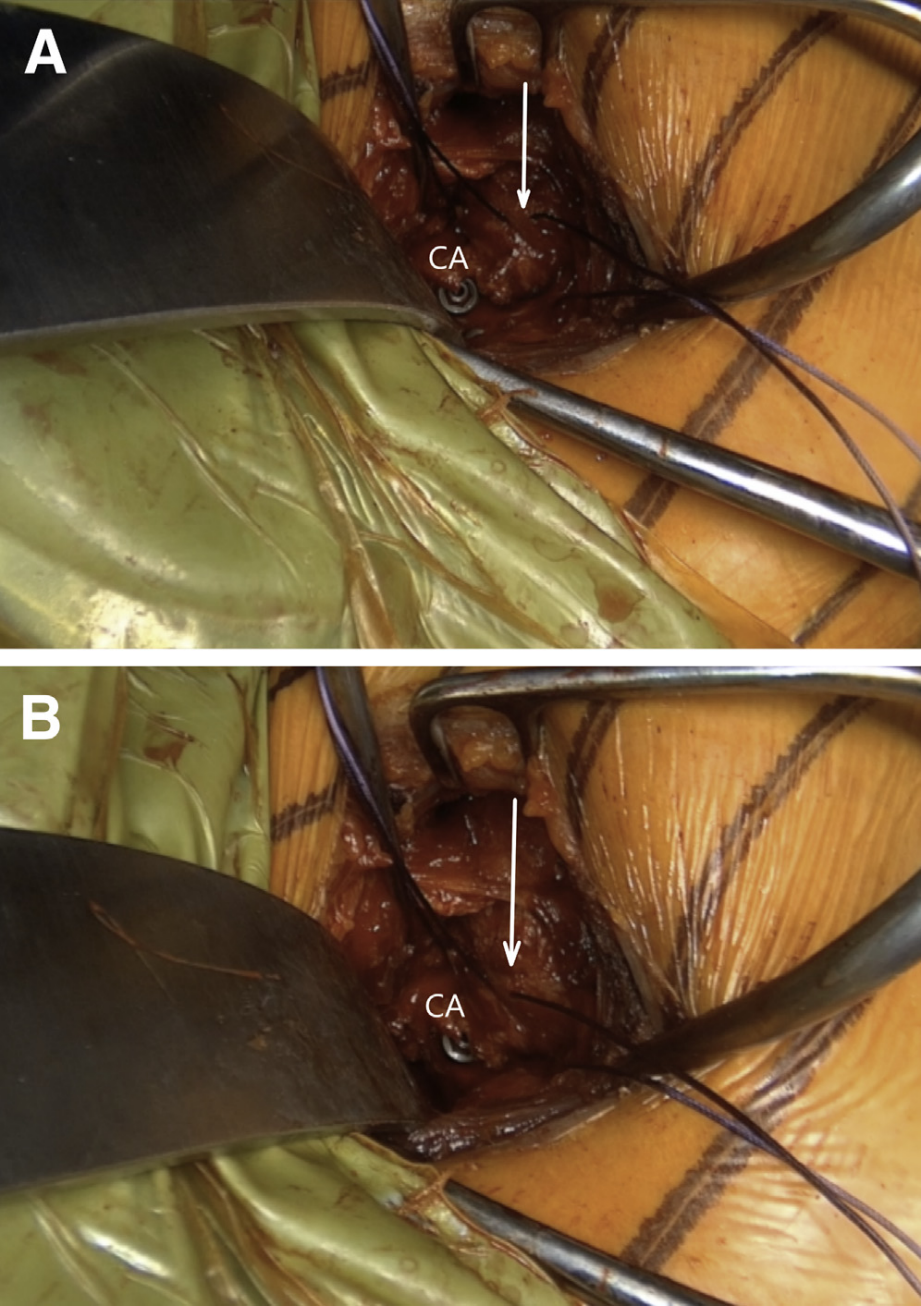
Left shoulder of a patient placed in a semi beach-chair position. Finally, the anterior capsule (white arrow) is reconstructed by the imbrication of the coracoacromial ligament (CA) with a resorbable suture. It is crucial that during the reconstruction, the arm is placed in adduction, anterior forward flexion, and external rotation and that (A) the anteriorly dislocated humeral head (B) is reduced.

Finally, the anterior capsule is reconstructed by the imbrication of the coracoacromial ligament with a resorbable suture. While the operated arm is held in external rotation to avoid the postoperative rotational deficit, the humeral head is reduced posteriorly in the center of the glenoid during adduction, slight anterior forward flexion, and a posterior lever push (Fig 19 and Video 1). Only then an adequate capsular tension is expected. The wound is copiously irrigated. The lateral tendinous part of the subscapularis is repaired with a nonresorbable suture. A standard layered closure is performed. Table 1 explains the pearls and pitfalls of this surgical technique.

**Table 1:** Pearls and Pitfalls


Pearls	When tightening the knots of the anterior capsule repair, we advise the surgeon to put the arm in slight abduction, approximately 45° of external rotation and reduce the humeral head to achieve adequate tension of the reconstructed anterior capsule.
Pitfalls	• Because of suture management during the Bankart repair and capsule reconstruction, the procedure is time-consuming. • The costs of the surgery would be greater when anchors would be used for Bankart repair and capsule reconstruction. • If the capsular repair is performed with the arm positioned in neutral or internal rotation, instead of the proposed 45° of external rotation, the risk of postoperative loss of external rotation is increased.

### Postoperative Rehabilitation

**Fig 20:**
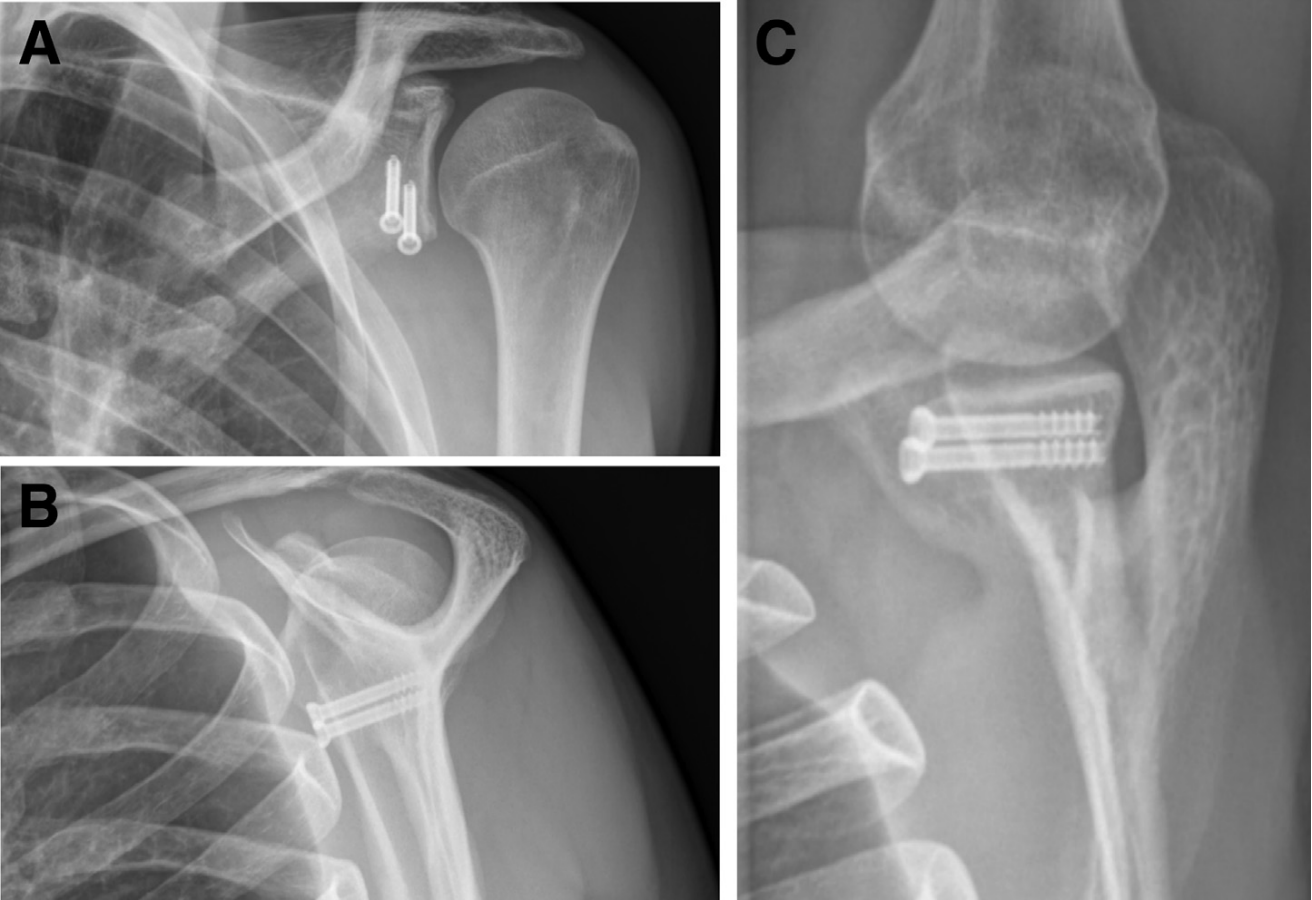
Anteroposterior, Neer, and Bernageau views of a left shoulder where a Latarjet procedure was performed. The coracoid graft is positioned flush with the glenoid face. Both screws are of the correct length and direction.

Three weeks of immobilization in a sling is recommended. On the first postoperative day, we allow complete passive range of motion. Three weeks after the surgery, the patient can commence with active range of motion. First follow-up with standard radiographs should be scheduled 6 weeks postoperatively (Fig 20 and Video 1).

## Discussion

The Latarjet procedure has a “triple-blocking effect” composed of 3 mechanisms that improve the anterior shoulder stability: first, the bony effect by bony coracoid block, which restores the glenoid bone loss and acts as a static restraint; second, the hammock and sling effects by conjoined tendon, which limits the anterior translation of the humeral head in a position of abduction and external rotation; and third, the bumper effect by reattachment of the anterior labrum and the capsule to the glenoid rim or coracoid process and reinforcement with coracoacromial ligament.^23^

It is unclear whether the labrum and the capsule should be reconstructed during the Latarjet procedure.^15^^,^^24^ However, it might have an important role. First, repairing the capsule to the anterior glenoid rim makes the coracoid block extra-articular, lowering the rate of dislocation arthropathy.^22^ Second, as shown in the cadaveric biomechanical study by Yamamoto et al.,^25^ the suturing of the capsular flap to the coracoacromial ligament contributed to 23% to 24% of shoulder stability at end-range arm position. In another biomechanical study, Kleiner et al.^26^ compared the effect of the Latarjet procedure with and without capsular-coracoacromial ligament repair. The augmentation of the Latarjet procedure with such capsular reconstruction showed a trend toward increasing anteroinferior translational stability in comparison with the Latarjet procedure alone. Itoigawa et al.^27^ showed that capsular repair to the glenoid rim tightens the anterior soft tissues, reducing external rotation but improving mid-range stability. In contrast, compared with capsular repair to the glenoid rim, capsular repair to the coracoid preserves external rotation without compromising end-range stability. With this in mind, it seems to us logical to preserve and repair the labrum to make the coracoid process extra-articular, then to perform a capsular repair to preserve external rotation and increase end-range stability^25^ while the mid-range stability is provided by the hammock and bone block effects.

Redislocation, persistent apprehension, postoperative limitation of external rotation, and development of glenohumeral osteoarthritis have long been described as one of the possible complications of the Latarjet procedure.^3^^,^^28^, ^29^, ^30^, ^31^, ^32^, ^33^ However, placing the arm in adduction, slight anterior forward flexion, and external rotation during the capsular reconstruction prevents the loss of external rotation.^27^ Besides the latter maneuver, we also routinely reduce the anteriorly subluxated/dislocated humeral head due to external rotation in our daily clinical practice while tightening the knots of the capsular reconstruction. This maneuver is similar to the posterior lever push used to improve visualization of the subscapularis during arthroscopic rotator cuff repair.^34^ We believe that this does not lead to restriction of the postoperative external rotation since the arm is also adducted and externally rotated. Moreover, we believe that due to the previously described posterior lever push maneuver, we avoid redundancy of the newly reconstructed anterior capsule and achieve its retensioning. The latter could lead to a greater degree of anterior stability of the humeral head and limits persistent micromotion previously reported.^35^ In addition, we perform a version of Bankart repair, where we repair the anteroinferior labrum to the coracoid process with the transosseous sutures that were shuttled through the coracoid graft. This results in extra-articular placement of the coracoid process, which might lead to a lower risk of osteoarthritis since the labral repair prevents the direct contact between the humeral head and the coracoid process.^20^^,^^22^ One of the authors (M.Z.) use, reinsertion of anchors for labral reconstruction.

Long-term consequences in the clinical setting need to be clarified by further long-term studies. Table 2 summarizes the advantages and disadvantages of this technique.

**Table 2:** Advantages and Disadvantages of the Proposed Technique


Advantages	• Appropriate tension of the newly reconstructed anterior capsule is achieved. • Because of associated Bankart repair, the rate of dislocation arthropathy is lowered. • Arthroscopic proficiency is not needed for this technique. • Allograft is not used, which lessens the expenses and increases the potential for graft integration with native glenoid.
Disadvantages	• Demanding surgical technique. • Anterior capsular reconstruction or Bankart repair can’t be performed in certain chronic cases where anterior capsule or anteroinferior labrum are inexistent or of very poor quality. • The surgeon needs to be careful during the preparation of the 2 coracoid holes intended for later labral fixation not to cause a fracture reaching toward the edge of the coracoid or towards the predrilled hole for later coracoid fixation. • The use of anchors for labral fixation carries the inherent risk of anchor migration.

## Conclusions

The presented technique of labral reinsertion on the glenoid, capsular and inferior glenohumeral ligament reattachment on the coracoacromial ligament after retensioning, and dynamic posterior glenohumeral reduction during soft-tissue reconstruction can adequately restore normal shoulder stability and additionally decrease postoperative microinstability, persistent apprehension, and risk of dislocation arthropathy.

## Supplementary Data

Video 1 After a 4- to 5-cm incision distal to the tip of the coracoid process, Richardson retractors are used to bluntly open the deltopectoral interval until the tip of the coracoid is exposed. A Hohmann retractor is placed on the base of the coracoid to gain its full exposure. The arm is put in abduction and full external rotation to improve visualization of the coracoacromial (CA) ligament, which is then released approximately 1.5 cm lateral from its attachment. Then, the pectoralis minor is released while the arm is internally rotated and adducted. A 90° angled saw blade is used to perform a coracoid process osteotomy just anterior to the attachment of coracoclavicular ligaments, and a chisel is meticulously used to complete the osteotomy. The coracoid process is rotated for 180° being held with a grasper and is attentively released until the muscle belly is uncovered. Its undersurface is first cleaned with electrocautery and is then flattened and slightly decorticated with a saw blade to create a healthy bleeding surface that will precisely conform to the later prepared anterior glenoid. The length of the coracoid is measured. Two central holes are drilled equally distant from the base and the tip, 1 cm apart and 8 to 9 mm laterally from the coracoacromial ligament insertion. Electrocautery is used to mark the entry holes. The coracoid is placed safely under the pectoralis major. The arm is put in abduction and external rotation. The subscapularis split between the upper two thirds and lower one third of the subscapularis is performed by sharply introducing the scissors toward the anterior glenoid neck. A Hohman retractor is placed between the blades on the medial side of the anterior glenoid neck. The subscapularis split is slightly increased laterally to improve visualization. A Gelpi retractor is placed deep below the subscapularis. The exact location of the glenohumeral joint is exposed by reducing the anteriorly dislocated humeral head, and a vertical incision is performed. A Trillat retractor is used next. The arm is put in adduction and internal rotation, and the Trillat retractor is introduced into the joint to get a better view of the anterior labrum. After the labrum is horizontally incised at 3 o’clock and released inferiorly, 2 sutures are passed through the released labrum. The Hohmann retractor is positioned on the inferior part of the glenoid neck, and a wide glenoid retractor is exchanged with the Hohmann retractor on the medial side of the anterior glenoid to improve the visualization. A curved osteotome is used to slightly decorticate the anteroinferior part of the glenoid. The position of the inferior hole is marked with a K-wire that is put 8 to 9 mm posteriorly to the glenoid face. The inferior screw is passed through the predrilled coracoid and glenoid hole to position the coracoid process on the anterior glenoid neck. The same is done for the superior screw. The screws are not fully tightened. A distinction between the CA ligament and the labrum, which is positioned posteriorly to the CA ligament, is made before the labrum is fixed on the coracoid process. Here, the Bankart repair was already performed. This is the CA ligament, and this is the anterior labrum that is fixed on the coracoid process. Then the sutures are passed through the CA ligament and through the anterior capsule. The operated arm is held in external rotation to avoid the postoperative rotational deficit. The humeral head is reduced posteriorly during adduction, slight anterior forward flexion, and a posterior lever push. At that time the knots of the anterior capsular reconstruction are tightened. Only then, an adequate capsular tension is expected. The lateral tendinous part of the subscapularis is repaired. The length of the incision ranges between 4 and 5 cm. A postoperative radiograph in the anteroposterior, Neer, and Bernageau views shows well-positioned graft and the 2 screws.

ICMJE author disclosure forms
